# Electron energy relaxation under terahertz excitation in (Cd_1−_*_x_*Zn*_x_*)_3_As_2_ Dirac semimetals

**DOI:** 10.3762/bjnano.8.17

**Published:** 2017-01-17

**Authors:** Alexandra V Galeeva, Ivan V Krylov, Konstantin A Drozdov, Anatoly F Knjazev, Alexey V Kochura, Alexander P Kuzmenko, Vasily S Zakhvalinskii, Sergey N Danilov, Ludmila I Ryabova, Dmitry R Khokhlov

**Affiliations:** 1 Physics Department, M.V. Lomonosov Moscow State University, Leninskie Gory 1 bld.2, 119991 Moscow, Russia; 2 Kursk Construction College, Sovetskaya str. 14, 305016 Kursk, Russia; 3 South-West State University, 50 Let Oktyabrya str. 94, 305040 Kursk, Russia; 4 Belgorod National Research University, Pobedy str. 85, 308015 Belgorod, Russia; 5 Faculty of Physics, University of Regensburg, Universitaetstr. 31, 93053 Regensburg, Germany,; 6 Chemistry Department, M.V. Lomonosov Moscow State University, Leninskie Gory 1 bld.3, 119991 Moscow, Russia

**Keywords:** Dirac semimetal, photo-electromagnetic effect, terahertz radiation, topological insulator

## Abstract

We demonstrate that measurements of the photo-electromagnetic effect using terahertz laser radiation provide an argument for the existence of highly conductive surface electron states with a spin texture in Dirac semimetals (Cd_1−_*_x_*Zn*_x_*)_3_As_2_. We performed a study on a range of (Cd_1−_*_x_*Zn*_x_*)_3_As_2_ mixed crystals undergoing a transition from the Dirac semimetal phase with an inverse electron energy spectrum to trivial a semiconductor with a direct spectrum in the crystal bulk by varying the composition *x*. We show that for the Dirac semimetal phase, the photo-electromagnetic effect amplitude is defined by the number of incident radiation quanta, whereas for the trivial semiconductor phase, it depends on the laser pulse power, irrespective of wavelength. We assume that such behavior is attributed to a strong damping of the interelectron interaction in the Dirac semimetal phase compared to the trivial semiconductor, which may be due to the formation of surface electron states with a spin texture in Dirac semimetals.

## Findings

The theoretical prediction of the existence of topological insulators [1–2] and their further experimental observation [3–7] have led to the significant growth of scientific interest in this area of research. Topological insulators form a class of materials possessing a different electron energy spectrum at the surface as compared to the bulk. The bulk energy spectrum of a topological insulator is typically observed for a semiconductor, whereas spin-polarized gapless electron states with a Dirac linear dispersion relation exist on the surface of a topological insulator.

Recently, another topological state of matter – a Dirac semimetal – was theoretically predicted [8] and then experimentally observed through the ARPES measurements in Cd_3_As_2_ [9–11]. In a Dirac semimetal, the bulk conduction and valence bands are inverted and touch each other at two points of the Brillouin zone, the Dirac points. The dispersion relation is linear in the proximity of the Dirac points in all three dimensions of the momentum space. The high Fermi velocity of the Dirac electrons is most likely the reason for extremely high values of the electron mobility observed in Cd_3_As_2_, which is up to 10^7^ cm^2^/(V·s) [9,11].

The question concerning formation of spin-polarized surface electron states in 3D Dirac semimetals remains open. According to [9,11], the surface states of Cd_3_As_2_ do not possess any spin texture. However, the authors do not exclude the possibility that the spin-polarized surface states may be formed, but the sensitivity of the method used does not allow discriminating these states.

In this paper, we show that there is an indirect confirmation for the formation of spin-polarized electron states with high mobility on the surface of the Dirac semimetal solid solutions (Cd_1−_*_x_*Zn*_x_*)_3_As_2_ with the inverted energy spectrum in the bulk.

Our experimental approach is based on measurements of the photo-electromagnetic (PEM) effect induced by terahertz laser pulses. Previously, it has been demonstrated that in the range of (Bi_1−_*_x_*In*_x_*)_2_Se_3_ solid solutions which exhibit a transition from the topological insulator phase with the inverted electronic energy spectrum at low *x* to the trivial insulator with the direct spectrum at higher indium content there is a substantial difference in the radiation power dependence of the PEM effect amplitude for the two phases [12]. In the trivial insulator case, the PEM effect amplitude is defined by the incident radiation power, irrespective of wavelength, whereas for the topological insulator phase, it depends on the number of incident radiation quanta per unit time. In this paper, we apply this approach to the solid solutions of Dirac semimetals (Cd_1−_*_x_*Zn*_x_*)_3_As_2_ undergoing an analogous transition from the inverted to the direct spectrum in the bulk at *x* > 0.08 [13].

The samples under study were single crystals of (Cd_1−_*_x_*Zn*_x_*)_3_As_2_ grown from the vapor phase. The sample composition was measured by energy dispersive X-ray analysis (EDA). According to the dataset available [13], the transition from the inverted electronic spectrum to the direct one occurs in (Cd_1−_*_x_*Zn*_x_*)_3_As_2_ at *x* ≈ 0.08. In view of that, we have selected three samples for detailed study. Two of them correspond to the inverted spectrum composition region *x* < 0.08, the third one has *x* = 0.25 and has a direct spectrum. The main electrophysical parameters of the samples are summarized in Table 1. All samples were of the n-type. The free electron concentration measured using the Hall effect did not change in the temperature range 4.2–300 K. The resistivity temperature dependence is typical for degenerate semiconductors: the resistivity decrease with decreasing temperature from 300 K to 20 K is followed by a further saturation at *T* < 20 K. Since the free electron concentration does not change, this resistivity variation is completely determined by the mobility temperature dependence. It is important to note that the free electron concentration in the samples studied differs by a factor of four. The low temperature mobility absolute values are very high and exceed 10^5^ cm^2^/(V·s).

**Table 1:** The values of resistivity, ρ, free electron concentration, *n*, and mobility, μ, for (Cd_1−_*_x_*Zn*_x_*)_3_As_2_ samples at *T* = 4.2 K.

*x*	ρ, mΩ∙cm	*n*, 10^17^ cm^−3^	μ, 10^5^ cm^2^/(V∙s)

0.012	0.064	1.8	5.65
0.045	0.157	4.0	1.01
0.25	0.061	3.4	3.05

The main tool of the study was the measurement of the PEM effect induced by terahertz laser pulses at the wavelengths 90 and 148 μm in a magnetic field up to 3 T. The pulse power was up to 10 kW, and the sample temperature was *T* = 4.2 K. The experimental details can be found elsewhere [12,14–19].

**Figure 1:**
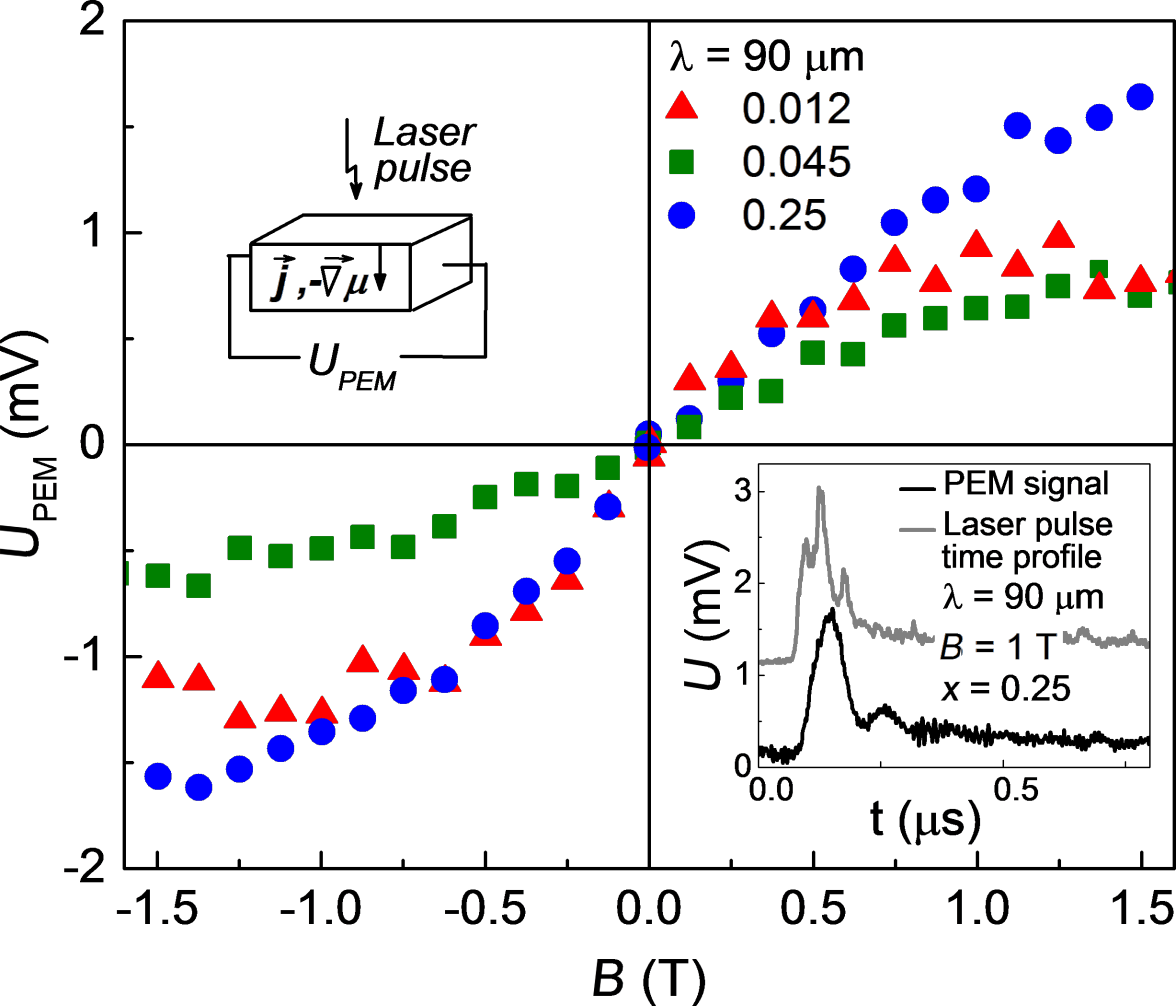
Dependence of the PEM effect amplitude on the magnetic field *B* applied for (Cd_1−_*_x_*Zn*_x_*)_3_As_2_ samples for alloy compositions *x* = 0.012, 0.045 and 0.25. The laser wavelength was λ = 90 μm and *T* = 4.2 K. The experimental geometry is shown in the upper left inset. Typical shape of the laser pulse and PEM response are shown in the lower right inset.

The PEM effect manifests itself as the appearance of a voltage decrease across the sample in the direction normal to the magnetic field and to the incident radiation flux. The experimental geometry is shown in the insert of Figure 1. The effect originates from the Lorentz force action to the diffusive electron flux which is due to excitation of the surface layer of a sample by the incident terahertz radiation. The voltage sign is defined solely by the direction of the net charge carrier flux either to or from the surface and does not depend on the charge carrier sign.

The PEM effect was observed in all samples at both laser wavelengths used. The kinetics of the PEM effect signal follow the signature of the time profile of the laser pulse (see insert in Figure 1). The effect occurs only under a nonzero magnetic field, and its amplitude is odd under the magnetic field. The magnetic field dependence of the PEM effect amplitude *U*_PEM_ is shown in Figure 1. For all samples, the effect is linear under low fields *B* < 1.5 T with a tendency for saturation under higher fields. It is important to note that the PEM effect sign corresponds to the net electron flux from the sample surface to its bulk.

**Figure 2:**
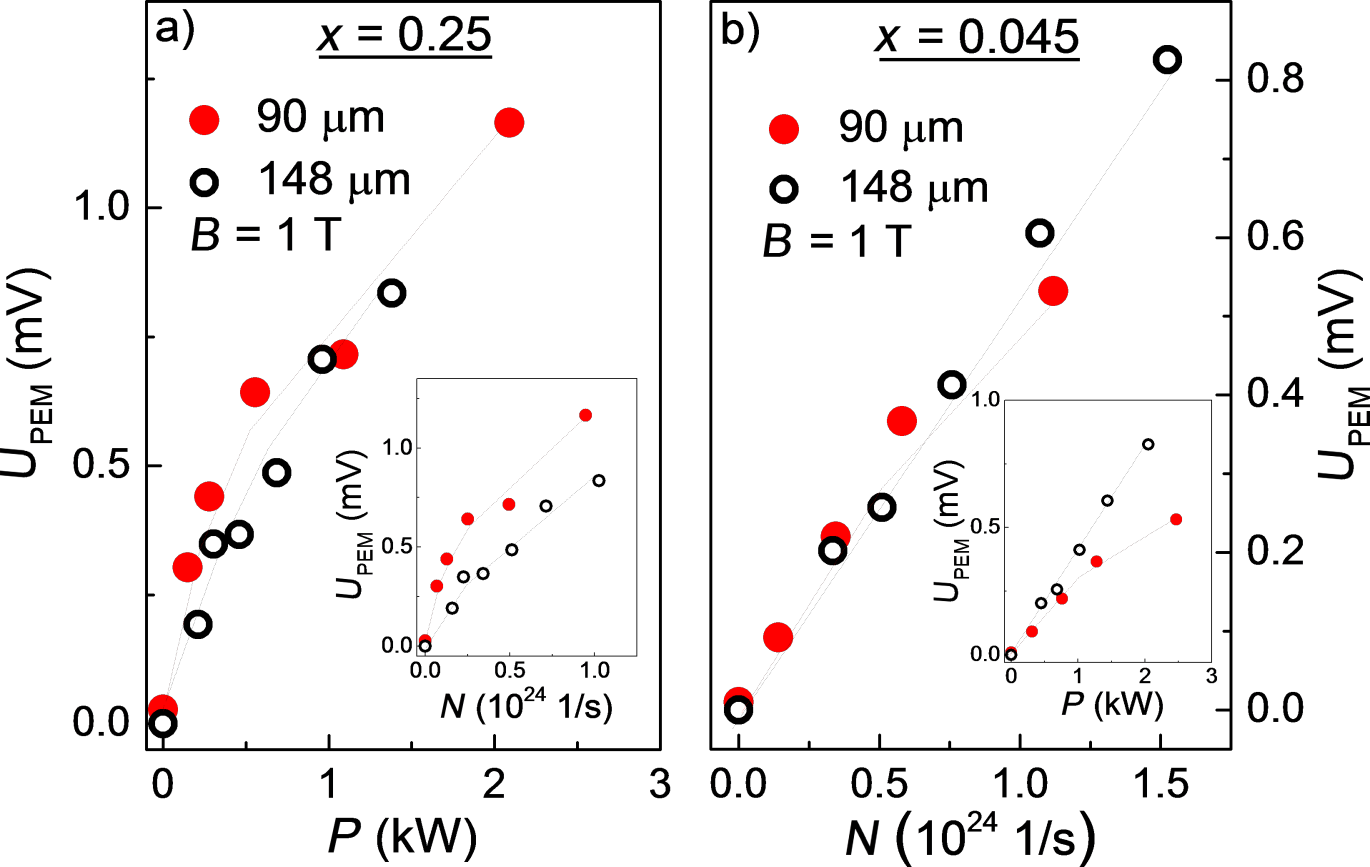
(a) Dependence of the PEM effect amplitude *U*_PEM_ on the incident laser radiation power *P* at *T* = 4.2 K for laser wavelengths 90 and 148 μm for a (Cd_0.75_Zn_0.25_)_3_As_2_ sample in trivial insulator phase. Inset: The same data plotted as PEM effect amplitude versus number of incident quanta *N*. (b) Dependence of the PEM effect amplitude on the flux of incident radiation quanta *N* at *T* = 4.2 K for laser wavelengths 90 and 148 μm for a (Cd_0.955_Zn_0.045_)_3_As_2_ sample in the Dirac semimetal phase. Inset: The same data plotted as PEM effect amplitude versus incident radiation power *P*. The lines in the panels (a) and (b) are the guides for the eye.

The effect of the radiation intensity variation on the PEM effect amplitude is shown in Figure 2. For the direct spectrum sample with *x* = 0.25 (Figure 2a), the effect scales up as a function of the incident radiation power for both laser wavelengths used (the main panel), whereas the dependence of *U*_PEM_ on the number of radiation quanta is different for different laser wavelengths (insert in Figure 2a). Instead, the inverse spectrum sample with *x* = 0.045 demonstrates a completely opposite behavior (Figure 2b): the *U*_PEM_ dependence on the number of incident quanta is the same for both wavelengths (the main panel), and the *U*_PEM_ dependence on the terahertz radiation power diverges for different wavelengths (insert in Figure 2b).

As it has been shown in [12,18], the diffusive electron flux from the surface to the bulk of degenerate semiconductors may arise only as a result of formation of conductive surface electron states with the mobility higher than in the bulk. These kind of surface states are apparently present both in the inverse and direct gap phases of (Cd_1−_*_x_*Zn*_x_*)_3_As_2_ solid solutions. The effect amplitude, however, scales up differently as a function of the incident radiation flux in the two cases. It was suggested in [12] that this difference in the case of topological insulators (Bi_1−_*_x_*In*_x_*)_2_Se_3_ is due to the different relation between the characteristic thermalization τ_th_ and diffusion τ_dif_ times of free electrons excited by the incident terahertz radiation. In the trivial insulator case, τ_th_ << τ_dif_, so the excited electrons first thermalize and then start to diffuse, thus the effect depends on the power absorbed. In the case of a topological insulator, the reverse relation τ_th_ >> τ_dif_ is realized, so the diffusion starts first, and then the number of diffusing electrons depends on the number of incident quanta. The thermalization of electrons excited by the incident terahertz radiation occurs mainly via the interelectron interaction since the optical phonon energy is higher than the laser quantum energy used. Consequently, the strong enhancement of the thermalization time in topological insulators compared to the trivial insulators is likely to result in the reduction of the number of electrons that interact effectively with a given one. As it was suggested in [12], this reduction may be due to the locking of the spin direction to the momentum direction of surface electron states in topological insulators. Therefore, the surface electrons may effectively interact only with other electrons possessing the same spin and, respectively, the momentum direction, and not with the whole Fermi sphere, as in the case of trivial insulators. Therefore, this enhancement of the thermalization time is an indication of the appearance of the spin texture of surface electron states. The results of the present experiment demonstrate that this kind of spin texture appears not only in topological insulators, but in the Dirac semimetals as well.

In conclusion, we have demonstrated through the measurements of the photo-electromagnetic effect that the thermalization time is strongly enhanced in the Dirac semimetals (Cd_1−_*_x_*Zn*_x_*)_3_As_2_, which is an indirect confirmation for formation of spin-polarized highly conductive electron states at their surface.
